# Evaluation of Silybin Nanoparticles against Liver Damage in Murine *Schistosomiasis mansoni* Infection

**DOI:** 10.3390/pharmaceutics16050618

**Published:** 2024-05-04

**Authors:** Daniel Figueiredo Vanzan, Ester Puna Goma, Fernanda Resende Locatelli, Thiago da Silva Honorio, Priscila de Souza Furtado, Carlos Rangel Rodrigues, Valeria Pereira de Sousa, Hilton Antônio Mata dos Santos, Flávia Almada do Carmo, Alice Simon, Alexandre dos Santos Pyrrho, António José Ribeiro, Lucio Mendes Cabral

**Affiliations:** 1 Department of Drugs and Pharmaceutics, Faculty of Pharmacy, Universidade Federal do Rio de Janeiro, Rio de Janeiro 21941-902, Brazil; danielfvanzan@gmail.com (D.F.V.); facarmo@pharma.ufrj.br (F.A.d.C.); 2 Department of Clinical and Toxicological Analysis, Faculty of Pharmacy, Universidade Federal do Rio de Janeiro, Rio de Janeiro 21941-902, Brazil; 3 Faculty of Pharmacy, University of Coimbra, 3000-548 Coimbra, Portugal; aribeiro@ff.uc.pt; 4 Group Genetics of Cognitive Dysfunction, I3S-Instituto de Investigação e Inovação em Saúde, Universidade do Porto, 4169-007 Porto, Portugal

**Keywords:** silybin, schistosomiasis, solid lipid nanoparticles, polymeric nanoparticles, polyphenolic compound

## Abstract

Silybin (SIB) is a hepatoprotective drug known for its poor oral bioavailability, attributed to its classification as a class IV drug with significant metabolism during the first-pass effect. This study explored the potential of solid lipid nanoparticles with (SLN-SIB-U) or without (SLN-SIB) ursodeoxycholic acid and polymeric nanoparticles (PN-SIB) as delivery systems for SIB. The efficacy of these nanosystems was assessed through in vitro studies using the GRX and Caco-2 cell lines for permeability and proliferation assays, respectively, as well as in vivo experiments employing a murine model of *Schistosomiasis mansoni* infection in BALB/c mice. The mean diameter and encapsulation efficiency of the nanosystems were as follows: SLN-SIB (252.8 ± 4.4 nm, 90.28 ± 2.2%), SLN-SIB-U (252.9 ± 14.4 nm, 77.05 ± 2.8%), and PN-SIB (241.8 ± 4.1 nm, 98.0 ± 0.2%). In the proliferation assay with the GRX cell line, SLN-SIB and SLN-SIB-U exhibited inhibitory effects of 43.09 ± 5.74% and 38.78 ± 3.78%, respectively, compared to PN-SIB, which showed no inhibitory effect. Moreover, SLN-SIB-U demonstrated a greater apparent permeability coefficient (25.82 ± 2.2) than PN-SIB (20.76 ± 0.1), which was twice as high as that of SLN-SIB (11.32 ± 4.6) and pure SIB (11.28 ± 0.2). These findings suggest that solid lipid nanosystems hold promise for further in vivo investigations. In the murine model of acute-phase *Schistosomiasis mansoni* infection, both SLN-SIB and SLN-SIB-U displayed hepatoprotective effects, as evidenced by lower alanine amino transferase values (22.89 ± 1.6 and 23.93 ± 2.4 U/L, respectively) than those in control groups I (29.55 ± 0.7 U/L) and I+SIB (34.29 ± 0.3 U/L). Among the prepared nanosystems, SLN-SIB-U emerges as a promising candidate for enhancing the pharmacokinetic properties of SIB.

## 1. Introduction

Silymarin (Sili) is an extract of a medicinal plant (*Sylibum marianum*) composed of flavonolignans, such as silycristin, silydianin, isosilybin, and silybin [1]. Silybin (SIB) comprises 50–70% of the Sili composition and is the principal active component. SIB is a drug that has antioxidant, anti-inflammatory, antifibrotic, and immunomodulatory properties [2,3]. The antioxidant effect occurs through direct or indirect effects on reactive oxygen species, stimulating production of endogenous antioxidant agents [4]. Its anti-inflammatory effect is related to the inhibition of the NF-κB pathway, which is responsible for initiating the transcription of genes associated with inflammation [5]. Furthermore, its anti-inflammatory effect is also associated with increased levels of IL-10, an anti-inflammatory cytokine [6]. Because of its antifibrotic property, its anti-inflammatory effect is related to a decrease in collagen synthesis and inhibition of the proliferation of activated hepatic stellate cells (HSCs) with a myofibroblast phenotype [7].

The hepatoprotective action of Sili and SIB on alcoholic [8,9] and nonalcoholic [10,11] liver diseases, hepatocellular carcinoma [12,13], and hepatitis C virus (HCV) infection has been reported [14]. In addition, Sili and SIB present hepatoprotective activity in liver diseases of parasitic origin, such as liver disease resulting from infection by *Schistosoma mansoni* [3,15]. The liver disease resulting from this infection occurs due to the obstruction of intrahepatic vessels by eggs of this parasite. These parasites (male and female) inhabit intestinal enteric vessels. The obstruction caused by eggs is due to oviposition by females at this location. These eggs can be carried by the hepatic portal circulation to the liver, where they cause tissue damage. In response to the presence of eggs in liver tissue, a periovular granulomatous inflammatory reaction is initiated. This reaction culminates in the formation of a fibrotic scar after the degradation of the egg. The accumulation of these scars will produce sequelae that remain even after the elimination of parasites through parasitological treatments. Among the sequelae caused by the accumulation of fibrosis in liver tissue, portal hypertension, hepatosplenomegaly, and esophageal varices are the most common reported [16,17].

The main cell type involved in fibrogenic processes in liver disease is myofibroblasts derived from HSCs. These cells reside in the space of Dissé between endothelial cells of hepatic sinusoids and hepatocytes [18]. When quiescent, HSCs can store lipids and vitamin A. These cells are responsible for storing 80% of the total vitamin A present in the body [16]. Activation of HSCs can occur through oxidative stress from reactive oxygen species, cellular components released by tissue necrosis, antigens, and stimulation of TGFβ1 and galectin 3 [19]. Upon activation, HSCs differentiate into myofibroblasts, losing the ability to store lipids and vitamin A to produce the extracellular matrix, particularly collagen types 1 and 3, for the formation of liver fibrosis [18]. In this context, Mata-Santos et al. [15] and Mata-Santos [3] demonstrated the antifibrotic, anti-inflammatory, antioxidant, and immunomodulatory effects of Sili intraperitoneally at a dose of 10 mg/kg on schistosomiasis in murine models of acute and chronic phases. In addition, Mata-Santos et al. [3] demonstrated the inhibiting action of Sili on the proliferation of myofibroblasts derived from HSCs, the murine cell line (GRX) cell line established by Borojevic et al. [20].

However, SIB has an absolute oral bioavailability of only 0.95% [21,22]. This is due to intense phase II metabolism, its low solubility in aqueous media, and its low permeability in biological membranes, and it is considered a Biopharmaceutics Classification System (BCS) class IV drug [21,22]. An alternative to improving the oral bioavailability of SIB is the use of technological strategies. Nanotechnology-based systems have been extensively explored to improve therapeutic efficacy and drug release properties while overcoming the obstacles of polyphenolic compounds, such as poor solubility and low oral bioavailability [23]. Solid lipid nanoparticles (SLNs) are colloidal nanocarriers that use solid lipids at room temperature as a method of drug encapsulation. The lipids used in this nanosystem, such as stearic acid, have high biocompatibility and are not toxic [24]. Other types of nanoparticles, such as polymeric nanoparticles (PNs), have shown great potential for targeted delivery to improve oral administration. PNs are solid systems that use a polymeric matrix, such as ε-polycaprolactone (PCL), to encapsulate drugs (nanocapsules) or form a polymeric core where drugs are retained or adsorbed (nanospheres), depending on the preparation method [25]. PCL is a biocompatible and biodegradable polymer widely used as a polymeric drug carrier [26].

During the development of nanosystems for gastrointestinal absorption, a mean diameter greater than 500 nm should be avoided, as these nanosystems begin to undergo phagocytosis by macrophages in situ, which will culminate in degradation and loss of part of the amount of drug administered [27,28]. Furthermore, the diameter range may influence the targeting and retention of nanosystems in liver tissue [27,29,30]. Blanco et al. [27] observed that nanoparticles >200 nm in size show greater accumulation and retention in hepatic tissue. He et al. [30] showed that nanoparticles with a size of 200–300 nm demonstrated greater intestinal transport and systemic biodistribution and greater targeting to hepatic tissue. These authors also demonstrated that sizes >300 nm, in addition to having less intestinal absorption, reach the plasma without hepatic targeting [30]. Griffin et al. [29] proposed that large nanoparticles (>300 nm) are more effective at targeting the lymphatic circulation and potentially avoiding first-pass hepatic metabolism. Accordingly, to increase intestinal absorption and hepatic targeting, the nanoparticles developed in this study should be between 200 and 300 nm in size.

Another alternative for directing nanosystems to therapeutic targets is modification of the surface of nanoparticles. The addition of acids and bile salts to the structure of nanoparticles can increase intestinal absorption via different mechanisms, in addition to promoting the targeting and accumulation of drugs in hepatic tissue. This phenomenon occurs through the cycle of bile acid and salt reuptake through the hepatic portal circulation to the liver [31]. Among the bile acids used, ursodeoxycholic acid (URSO) stands out for having a previously described hepatoprotective action [32,33,34,35]. URSO is a secondary bile acid derived from the metabolism of the intestinal microbiota [33]. Among bile acids, URSO is the most hydrophilic [31]. It has an anti-inflammatory effect by inhibiting the synthesis of hydrophobic bile acids, such as deoxycholic acid and lithocholic acid, protecting hepatocytes from the cytotoxic effects of these bile acids [36,37]. In addition, URSO has an antifibrotic effect through the inhibition of autophagy, which facilitates HSC activation [35]. In addition to its hepatoprotective effect, URSO can act as a permeability promoter, as ascribed to other bile acids. Permeation-promoting mechanisms are attributed to increased solubility of lipophilic drugs, opening of tight junctions, inhibition of proteolytic enzymes, and inhibition of efflux pumps by inhibiting P-glycoprotein [31].

In this study, SIB containing SLNs (with and without URSO) and PNs (with ε-polycaprolactone used as the polymeric matrix) were prepared and characterized. SLNs and PNs were first valuated through in vitro release studies, in vitro permeability in Caco-2 cells, and cell viability assays in GRX cells to investigate the effects of the nanoencapsulated SIB on the proliferation of these cells. After selecting the best nanosystem, in vivo studies were carried out in a murine model of *Schistosoma mansoni* infection in the acute phase.

## 2. Materials and Methods

### 2.1. Materials

Stearic acid was purchased from Exodo Cientifica (São Paulo, Brazil). ε-Polyprolactone (PCL), polyvinyl alcohol (PVA), a dialysis membrane with a pore size of 14,000 kDa, 3-(4,5-dimethyl-2-thiazolyl)-2,5-diphenyl-2H-tetrazolium bromide, chloramine T, p-dimethylaminobenzaldehyde (DMAB), Dulbecco’s modified Eagle’s medium, Hank’s balanced salt solution (HBSS), N-acetyl-L-cysteine (Nac), silymarin (Sili), silybin (SIB) (Figure S1A), and ursodeoxycholic acid (URSO) (Figure S1B) were purchased from Sigma-Aldrich Brasil Ltd. (São Paulo, Brazil). A Biomax^®^ ultrafiltration disc (100 kDa) was purchased from Merck Millipore Brasil Ltd. (São Paulo, Brazil). Potassium chloride, sodium hydroxide, hydrochloric acid, acetic acid, n-propanol, phosphoric acid, citric acid, sodium acetate trihydrate, potassium hydroxide, and Tween 20 were purchased from VETEC^®^ Química Fina Ltd. (São Paulo, Brazil). Acetone, acetonitrile, chloride sodium, sodium phosphate dibasic anhydrous, and potassium phosphate monobasic anhydrous were purchased from Neon Reagentes Químicos Ltd. (São Paulo, Brazil). Dimethylsulfoxide (DMSO) was purchased from Biograde Brasil Ltd. (Goiás, Brazil). Uranyl acetate aqueous solution (5%) was kindly provided by the Padrón-Lins Multi-User Microscopy Unit (UniMicro) at the Universidade Federal do Rio de Janeiro (Rio de Janeiro, Brazil). A 96-well cell culture plate was purchased from Kasvi^®^ Ltd. (Paraná, Brazil). Twelve-well transwell plates were purchased from Corning^®^ Ltd. (New York, NY, USA). Fetal bovine serum and penicillin–streptomycin (10,000 U/mL) were purchased from Gibco Scientific (New York, NY, USA). Commercial kits for lactate dehydrogenase (LDH), alanine aminotransferase (ALT), and aspartate aminotransferase (AST) were purchased from Labtest Diagnóstica S. A. (Minas Gerais, Brazil).

### 2.2. Preparation of SLN

Initially, the emulsification/evaporation/solidifying method [21,38] was used, and minor modifications were made to obtain a suitable nanosystem. Modifications were performed regarding ultrasonication time, surfactant proportion, lipid matrix, and SIB (Table 1). The first emulsion was obtained by dripping the organic phase into 30 mL of an aqueous phase containing a surfactant (1.30 or 1.67% (*w*/*v*) P20) using automatic dripping (Cole Parmer, IL, USA) at a rate of 10 mL/hour under fast magnetic stirring at 80 ± 2 °C. The organic phase consisted primarily of acetone and stearic acid, with or without the addition of URSO and SIB. Then, the lipid emulsion was concentrated by rotary evaporation (IKA^®^ RV 10 basic, Staufen, Germany) at 80 °C to eliminate the organic solvent and reduce the volume of the aqueous phase. Then, cold purified water (2–5 °C) was added until a volume of 13 mL was reached. The lipid emulsion system was quickly placed in an ice bath under fast stirring using ultrasonication (UP100H, Hielscher, NC, USA—cycle 1 and amplitude 100%) for 30 to 50 min to complete the process. The obtained SLNs were identified from positions 1 to 8 and evaluated for mean diameter, polydispersity index (PDI), and zeta potential using the Statistica^®^ program version 14.0.0 (TIBCO^®^, Palo Alto, CA, USA). SLNs with suitable characteristics (SLN-8) were subjected to ultrafiltration using an Amicon Model 8050 ultrafiltration cell (Merck KGaA^®^, Darmstadt, Germany) with a 100 kDa polyethersulfone Biomax^®^ membrane (Millipore, Billerica, MA, USA) and a vacuum pump. The nanosystem was subsequently rinsed seven times with the same volume (30 mL) of purified water to remove any residual organic solvent or nonencapsulated components from the nanosystem [39]. SLNs were prepared and immediately characterized. SLNs prepared for in vitro studies in cell culture and in vivo studies were refrigerated (4 ± 0.2 °C) for 72 h.

### 2.3. Preparation of PNs

PNs were prepared by the nanoprecipitation method [40]. The PCL polymer (60 mg) and SIB (10 mg) were dissolved in 5 mL of acetone using magnetic stirring. The aqueous phase was prepared by adding 0.1% polyvinyl alcohol (PVA) to 60 mL of purified water. Then, the organic phase was added to the aqueous phase using automatic dripping at a rate of 20 mL/hour (Cole Parmer, IL, USA) under magnetic stirring at 25 °C. The system was ultrasonicated with an amplitude of 100% and cycle 1 (UP100H, Hielscher, NC, USA) in an ice bath. After this step, the nanosystems were transferred to a rotary evaporator (IKA^®^ RV 10 basic) for 2 h at 100 rpm to evaporate the organic solvent. At the end of the process, the nanosystems were collected by serial ultracentrifugation (Hitachi^®^ CR22GIII, Tokyo, Japan) at 5000, 10,000, and 20,000 rpm for 30 min at 25 °C, followed by pellet resuspension in purified water. PNs were prepared and immediately characterized.

### 2.4. Physicochemical Characterization of the Nanoparticles

#### 2.4.1. Mean Diameter, Polydispersity Index, Zeta Potential, and Morphology

The mean diameter and polydispersity index (PDI) were determined by dynamic light scattering (Zetasizer Nano S90^®^, Malvern Instruments, Malvern, UK). The viscosity (0.88732 cp) and refractive index (1.33) of water were used. The nanosystems were diluted in distilled water at 25 °C (1:3) to decrease the opacity, and average values were obtained from ten consecutive measurements of the same sample in triplicate. The zeta potential was evaluated by the phase analysis light scattering technique using a NanoBrook ZetaPALS Potential Analyzer^®^ (Brookhaven Instruments Co., Holtsville, NY, USA). For this analysis, the nanosystems were diluted in purified water (1:10) and then transferred to a dip cell-type cuvette (1.5 mL) in which an electrolytic cell was introduced. The analysis was performed in triplicate with ten measurements per sample using the measured mean particle diameter and the pH of the formulations as inputs. The morphological characteristics of the nanosystems were evaluated by transmission electron microscopy (TEM) using an FEI Tecnai^TM^ Spirit 120 Kv microscope (Hillsboro, OR, USA) at CENABIO. A 10 µL volume of each nanosystem was placed onto 300 mesh formvar/carbon-coated copper grids for 2 min. Then, the grids were dried with filter paper, and for background contrast, 5% (*w*/*v*) uranyl acetate was added. Subsequently, the prepared grids were observed via TEM [41,42].

#### 2.4.2. SIB and URSO Quantification

SIB and URSO were quantified using a high-performance liquid chromatograph equipped with a diode array detector (HPLC-DAD) (Shimadzu Corporation, Kyoto, Japan; LC-30AD pump, SIL-30AC autosampler, CTO-20A column oven, SPD-M20A detector). The proposed chromatographic conditions were based on the method described by Khairy and Mansour [43] for URSO quantification; however, the method was optimized for the simultaneous quantification of SIB and URSO. Chromatographic separation was achieved isocratically at 40 °C using a Shim-pack GIST C18 column (150 × 4.6 mm; 5 μm) with a mobile phase composed of 100 mM phosphate buffer (pH 3.0), acetonitrile (1:1, *v*/*v*), a flow rate of 0.5 mL/min, and an injection volume of 30 μL. SIB and URSO were detected by ultraviolet light at a wavelength of 210 nm. The SIB stock solution was prepared by accurately weighing 5 mg and dissolving it in 25 mL of acetonitrile. The URSO stock solution was prepared by accurately weighing 10 mg, dissolving 0.5 mL of DMSO, and increasing the volume of acetonitrile to 10 mL. The linearity curve was prepared from serial dilutions in triplicate. Chromatography was carried out to quantify the encapsulation efficiency, in vitro release, and cell permeability of the samples. For the determination of the encapsulation efficiency, a linear curve was prepared with 5 concentrations, between 20 and 80 µg/mL for SIB and 100 and 400 µg/mL for URSO. In the in vitro release test and cell permeability assay, the linearity curve was prepared in the range of 0.1–30 µg/mL for SIB, while the low UV absorption of the URSO molecule rendered it impossible to quantify URSO. The linearity curves were prepared from serial dilutions of the stock solution in triplicate. The HPLC method was partially validated by evaluating parameters such as selectivity, linearity, quantification, and detection limits [44].

#### 2.4.3. Process Yield and Encapsulation Efficiency (EE)

The process yield (%) was evaluated after freeze-drying the nanosystems using an L101 lyophilizer (LÍOTOP, Barra Mansa, Brazil). The yield (%) was calculated from the ratio between the freeze-dried nanosystem mass and the initial mass of the total components used in the nanosystem [41,42]. The EE percentage (%) of SIB and URSO was determined using an indirect method after the ultrafiltration step for the SLNs and after the ultracentrifugation step for the PNs using the HPLC method described above. The EE% was determined using the frequently described equation [41,42].

#### 2.4.4. SIB Release Studies

First, the solubility of the SIB was evaluated in two biorelevant receptor media, simulated gastric fluid (SGF, pH 1.2) supplemented with 2% P20 and phosphate-buffered saline (PBS, pH 7.4) supplemented with 1% P20, using the shake-flask method [41,45]. An excess amount of SIB was added to 1.5 mL of each medium at 37 °C, which was kept under magnetic stirring (150 RPM) for 24 h, followed by centrifugation at 7000 rpm for 15 min. The supernatant was filtered through a 0.45 μm hydrophobic polyvinylidene fluoride (PVDF) membrane and then quantified using HPLC. In vitro release studies of SIB encapsulated in nanosystems were carried out by the dialysis bag method using a dialysis tubing cellulose membrane (33 mm; 14,000 g·mol^−1^) (Sigma-Aldrich, St. Louis, MO, USA) [41,46,47]. The dialysis bags containing the nanosystems (6.5 mL) were immersed in a vessel containing 250 mL of biorelevant medium at 37 °C under magnetic stirring at 150 rpm for 3 h in SGF 2% P20 medium and for 48 h in PBS 1% P20 medium. The experiment was performed in triplicate, and at different time intervals—0.25, 0.5, 0.75, 1.0, 2.0, and 3.0 h for SGF 2% P20 media and 0.25, 0.5, 1.0, 2.0, 3.0, 4.0, 5.0, 24, and 48 h for PBS 1% P20 media—5 mL from the receptor media was withdrawn and then replenished with fresh media to maintain the sink conditions. Subsequently, the SIB present in the collected aliquots was quantified using HPLC. The results are expressed as the cumulative amount of SIB released (%) against time (h).

The flow through the membrane (*J*) was determined from the slopes of the linear portions of the curves, and the lag time (*tlag*) was calculated by extrapolating the steady-state slope to its intersection on the time axis. Kinetic analysis was achieved by applying zero-order, first-order, Higuchi, and Korsmeyer–Peppas kinetic models to evaluate the mechanism of SIB release from nanosystems [41].

#### 2.4.5. Stability Study

A stability study was performed under refrigeration (4 ± 0.2 °C) and at room temperature (25 ± 0.5 °C) for up to 120 days for the SLNs group. The physical–chemical characteristics, such as the mean diameter and PDI, were evaluated using the methods described above.

### 2.5. In Vitro Studies in Cell Culture

Two immortalized cell lines were used. A myofibroblast line extracted from murine hepatic granulomas induced by S. mansoni infection, GRX cells, was used for cell proliferation and viability assays. A human colon adenocarcinoma cell line (Caco-2) was used to determine the permeability coefficient across the intestinal barrier. GRX and Caco-2 cells were cultured at 37 °C in 5% CO_2_ and 95% atmospheric pressure in Dulbecco’s modified Eagle’s medium (DMEM) supplemented with 10% fetal bovine serum (FBS).

#### 2.5.1. Cell Proliferation and Viability Assays

First, cell viability was assessed using 3-(4,5-dimethyl-2-thiazolyl)-2,5-diphenyl-2H-tetrazolium bromide (MTT) to assess the concentrations of SIB, Sili, and nanosystems suitable for cell proliferation assays.

For the lactate dehydrogenase (LDH) and cell proliferation assays using MTT, 1 × 10^4^ GRX cells/well were seeded in 96-well plates and incubated for 24 h. Then, the cells were exposed to different concentrations of SIB, Sili, or the nanosystems for 24, 48, and 72 h. The concentrations of 25, 50, and 100 µM Sili and SIB prepared in DMEM containing 0.5% (*v*/*v*) dimethyl sulfoxide (DMSO) were evaluated. The SLN-SIB, SLN-SIB-U, and PN-SIB nanosystems were evaluated at concentrations of 350 and 700 nM, as well as their controls (SLNs and PNs), which were all prepared in DMEM. In addition, the effects of the diluent (DMEM/0.5% DMSO), the growth control (DMEM), and the antioxidant control on cell growth inhibition, N-acetyl-L-cysteine (Nac), at a concentration of 10 mM were evaluated. The cell membrane integrity was evaluated by quantifying LDH via a commercial cytotoxicity detection kit following the manufacturer’s instructions using a microplate spectrophotometer (SpectraMax^®^ I3, Molecular Devices, CA) with an absorbance of 340 nm. The cell proliferation assay was based on the cleavage of the tetrazolium salt MTT (0.5 mg/mL) after incubation for 3 h, using dimethyl sulfoxide (DMSO) to dissolve formazan crystals quantified by a microplate spectrophotometer with an absorbance of 570 nm [3]. The inhibitory effects of Sili, SIB, SLN-SIB, SLN-SIB-U, PN-SIB, and NAC were calculated in relation to their respective growth controls (0.5% DMSO, SLN, PN). The inhibitory effect was calculated using the equation as follows:

$$\mathrm{Inhibitory}\;\mathrm{effect}\;\left(\%\right)=100-\left(\frac{\%\;\mathrm{growth}\;\mathrm{substance}\;\mathrm{or}\;\mathrm{nanoparticle}}{\%\;\mathrm{growth}\;\mathrm{vehicle}}\times 100\right)$$

#### 2.5.2. Permeability Assay on Caco-2 Monolayer

First, cytotoxicity testing using the MTT assay was carried out to investigate the effect of the concentrations of SIB, Sili, and nanosystems on Caco-2 cell viability. Caco-2 cells were seeded (1 × 10^5^ cells/200 µL/well) in DMEM supplemented with 10% FBS at 37 °C in 5% CO_2_ and 95% atmospheric pressure. After 24 h, the culture medium was removed from the wells, and the cells were placed in contact for 3 h with the samples at concentrations of 25 and 80 µM for Sili and SIB and 12 and 40 µM for the nanosystems diluted in medium or Hank’s balanced salt solution (HBSS). DMEM and 1% (*v*/*v*) DMSO were used as control groups. Then, the samples were aspirated, the cells were treated with MTT solution (0.5 mg/mL) as described previously, and cell viability was evaluated.

For the permeability assay, Caco-2 cells (density of 5.0 × 10^5^ cells/cm^2^) were seeded in Transwell^®^ filters with a pore size of 0.4 µm, a diameter of 12.0 mm, and an area of 1.12 cm^2^ using a 12-well plate and culture medium. After 21 days, monolayer integrity was confirmed by monitoring transepithelial electrical resistance using a Millicell^®^ ERS-2 Voltohmmeter (Millipore Corp., Burlington, MA, USA) [47]. The monolayer integrity was considered when voltages above 400 Ω.cm^2^ were observed. Afterward, 500 µL of sample (25 µM Sili, SIB, or nanosystems) prepared in HBSS was placed in the apical compartment, and 1500 µL of HBSS was placed in the basolateral compartment. At fixed times (30, 60, 90, and 120 min), 1500 µL from the basolateral compartment was collected and quantified using the HPLC method described above. The same volume was replaced with fresh HBSS at each collection time. The permeability assay was performed in triplicate, and the integrity of the monolayer was checked at the start and end of the assay. All samples collected from the basolateral compartment were quantified using the HPLC method described above. The apparent permeability coefficient (*Papp*) was calculated from the following equation:

$$Papp=\frac{dQ}{dt\;\;}\frac{1}{AC_{0}}$$

where *Papp* is the apparent permeability coefficient, *dQ*/*dt* is the flux through the cell monolayer, *A* is the surface of the monolayer in cm^2^, and *C*_0_ is the initial concentration of the drug in the apical compartment [21,47].

### 2.6. In Vivo Studies

#### 2.6.1. Animals and Experimental Model

The animal model used for the study was 6–8-week-old female BALB/c mice. The animals were kept in microisolator cages and fed a complete diet for rodents and water ad libitum, on a 12/12 h light/dark cycle. All experimental procedures were approved and conducted in accordance with guidelines for the care and use of laboratory animals (CEUA) from the Centro de Ciências da Saúde (CCS) of the Universidade Federal do Rio de Janeiro (protocol number 115/22), which conform to the National Institute of Health (Bethesda, MD, USA) guidelines.

The murine experimental model of infection by the *Schistosoma mansoni* BH strain followed that described by Mata-Santos [3,15]. Prior to the experiments, some of the animals were kept without infection, and others were infected with 80 cercariae of *Schistosoma mansoni* (BH strain) via the cutaneous route (5 mL/40 min). Animals were then monitored for 35 days postinfection (35 dpi). On 35 dpi, noninfected (N) controls and infected (I) animals were divided randomly into nine groups of 3 animals (Table 2). Then, the animals were submitted to oral treatment through gavage of the SLN, SLN-SIB, and SLN-SIB-U nanosystems (1 mg/kg/day) and SIB in 0.1% carboxymethylcellulose (10 mg/kg/day) for 30 consecutive days. In the control group, N and I animals were treated with purified water at the same time. Animals from all groups were maintained under controlled temperature and light conditions and were fed a balanced diet and sterile water ad libitum. After 66 dpi, the animals were subjected to euthanasia under anesthesia/cervical dislocation. Serum samples and liver, spleen, and intestine samples were removed for evaluation.

#### 2.6.2. Relative Liver Weight

The effect of nanosystems on liver and spleen enlargement in the murine schistosomiasis mansoni model was calculated from the percentage of the ratio between the liver or spleen weight/animal body weight.

#### 2.6.3. Parasitological Evaluation

Hepatic and intestinal tissues were digested as previously described by Cheever [48]. In brief, tissues were maintained in 4% KOH at room temperature for 12 h, followed by 3 h of incubation at 37 °C and microscopic evaluation. The results are expressed as eggs per gram of liver. Eight independent samples were counted.

#### 2.6.4. Dosage of Liver Enzymes Levels

The ALT and AST levels in the serum, which are markers of hepatocellular damage, were measured via a colorimetric assay using a commercial ALT/GPT Liquiform 108 and AST/GOT Liquiform 109 kit, respectively, following the manufacturer’s instructions (Labtest Diagnostic, Lagoa Santa, Brazil).

#### 2.6.5. Hydroxyproline Determination

Hydroxyproline quantification was carried out based on a previously reported methodology [49,50]. Briefly, livers were maintained in acetone at room temperature until complete dehydration, followed by hydrochloric acid hydrolysis overnight at 107 °C. A colorimetric assay was then performed using a buffered Chloramine T solution (Sigma-Aldrich, St. Louis, MO, USA), Ehrlich’s aldehyde reagent (Sigma-Aldrich, St. Louis, MO, USA), and perchloric acid (Merck, Rio De Janerio, Brazil). The results are expressed as milligrams of hydroxyproline per gram of liver tissue [15,49,51]. Groups were represented by three independent pools of livers, each composed of three organs.

### 2.7. Statistical Analysis

The results are expressed as the mean ± standard deviation (SD). Differences between the mean values of the control and experimental groups were assessed using one-way analysis of variance (ANOVA), followed by Tukey’s test. Statistical analyses were performed using Prism software (GraphPad Software Inc., Irvine, CA, USA, https://www.prismsoftware.com/), and a *p* value ≤ 0.05 was considered to indicate statistical significance.

## 3. Results

SIB has low water solubility and permeability in biological membranes; consequently, it has low oral bioavailability and is subject to extensive first-pass metabolism. To improve the oral bioavailability of SIB, for this study, two nanosystems were proposed: solid lipid nanoparticles obtained by a modified emulsification/evaporation/solidification method and polymeric nanoparticles obtained by a nanoprecipitation method.

### 3.1. Evaluation of the Preparation Method of SLNs Containing SIB

The initial screening of lipid nanoparticles was based on obtaining nanoparticles with a mean diameter between 200 and 300 nm and a PDI less than 0.3, which is considered acceptable and indicates a homogenous population of phospholipid vesicles [52]. In this sense, the emulsification/evaporation/solidifying preparation method was selected, and minor modifications, such as ultrasonication time, surfactant proportion, lipid matrix, and SIB, were applied (Table 1) with the aim of obtaining suitable nanoparticles. The results of the physicochemical characterization of the SLNs obtained using different preparation methods are described in Table 3.

The preparation method “A” followed that described by Piazini et al. [21]. These authors prepared SLNs that exhibited an average diameter less than 170 nm and good properties in terms of homogeneity, with a PDI less than 0.3. However, a mean diameter > 400 and PDI greater than 0.4 were observed (Table 3). This fact can be attributed to the physicochemical properties of the surfactant used and indicates the need for modifications to the method. Commonly, the development of pharmaceutical products or methods has been carried out by analyzing one factor at a time; however, the design of experiments (DoE) may provide better results with few experiments [53]. The impact of the modifications on the method and composition of the nanosystems was assessed through analysis of Pareto diagrams (Figures S2 and S3; Supplementary Materials). The studies revealed a significant impact resulting from an increase in sonication time and surfactant amount, which was inversely proportional to the particle size and PDI value.

Methods “B”, “C”, and “D” proposed an increase in surfactant (1.30 to 1.67%) and a gradual increase in ultrasonication time (30, 40, and 50 min). According to Table 3, a longer sonication time and an amount of 1.67% surfactant were able to provide nanoparticles between 200 and 300 nm in size with a PDI less than 0.2. This phenomenon differs from that found by Badawi et al. [54], where improvements in these critical quality values were obtained with shorter ultrasonication times (10 min).

The addition of increasing amounts of URSO between 10 and 50 mg (*w*/*w*) and the consequent reduction in the amount of stearic acid in the nanosystem were also evaluated. The results showed that SLN-5 to SLN-8 presented a mean diameter of 240 to 290 nm, with a PDI less than 0.26, and there was no negative influence of the amount of URSO (Table 3). This event is favorable for the development of nanosystems since greater amounts of this bile acid can promote increased intestinal permeability and hepatic targeting. In addition, the 50 mg concentration is above the critical micellar concentration necessary for the formation of micelles [31]. Furthermore, the addition of 50 mg of URSO can increase the stability of the nanosystem.

After selection of the method and composition of the SLNs, an ultrafiltration procedure was performed to remove excess surfactant and other components that were not incorporated into the SLNs. The mean diameter and PDI of nanoparticles of the same composition obtained without (NP-4 and NP-8) and with ultrafiltration (SLN-SIB and SLN-SIB-U) were not significantly different (*p* < 0.05). The fact that ultrafiltration did not promote changes in particle size or PDI is favorable since this procedure is intended to remove components that are not part of the nanosystem [39]. Furthermore, using ultrafiltration, an increase in the modulus of zeta potential of SLN-SIB and SLN-SIB-U was observed. This fact indicates that the excess surfactant interfered with the surface charge of the particle.

### 3.2. Physicochemical Characterization of SLN and PN Containing SIB

#### 3.2.1. Mean Diameter, Polydispersity Index, Zeta Potential, and Morphology

SLNs containing SIB, SLNs containing URSO/SIB and PNs containing SIB were developed and physicochemically characterized (Table 4). There was a significant increase in the mean diameter and PDI after the incorporation of SIB and URSO/SIB in relation to those of the empty SLNs (*p* > 0.05). For PNs, the PN-SIB value was lower than that for PNs (*p* < 0.01). This variation indicates the possible interference of the addition of SIB and/or SIB/URSO to the nanosystems. However, the values for all nanosystems are below 500 nm. Furthermore, a PDI less than 0.3 indicates greater homogeneity of the nanosystems. Nanoparticles with sizes greater than 500 nm are expected to act upon macrophages in the gastrointestinal tract through phagocytosis [55], while nanoparticles smaller than 500 nm have greater intestinal absorption [21] and therefore greater bioavailability. In addition, a PDI less than 0.3 indicates greater homogeneity for nanosystems [52].

Regarding the zeta potential values, no variation was observed with the addition of SIB to SLNs, indicating that the presence of SIB does not interfere with the surface charge of the particles. However, the addition of URSO promoted a slight decrease in this value, indicating that the presence of this substance slightly interfered with the surface charge of the SLNs. However, the zeta potential values for all SLNs indicate acceptable stability due to repulsive forces between particles [56,57]. For PNs and PN-SIB, the zeta potential tends toward neutrality, but due to the addition of a dispersing agent (PVA), there is no need to use electrostatic interactions to maintain the stability of the particles [58].

The EE% values of SLNs and PNs were high, particularly for PN-SIB, which presented a value of approximately 100%. High EE% values culminate in greater drug transport through the gastrointestinal tract, increasing the amount of drug absorbed through the oral route. The mass yield was only high for PNs (>86%). On the other hand, the mass yield values of SLNs were lower (<40%). The differences in process yield can be attributed to the characteristics of the preparation and separation methods used to obtain the nanosystems. The emulsification/evaporation/solidifying method employed for SLNs requires a larger amount of surfactant to stabilize the droplets during preparation [21,38], whereas the nanoprecipitation method utilized for PNs requires a smaller amount of surfactant [40]. Regarding the separation techniques employed, SLN purification involves ultrafiltration to eliminate excess surfactant and other nonincorporated components [59]. The ultrafiltration method is applied due to the electrostatic interactions governing the stabilization of this nanosystem [60]. Notably, after ultrafiltration, a decrease in zeta potential values from −12 mV to −30 mV was observed (Table 3), indicating the removal of a substantial portion of the surfactant. This stage might account for the significant reduction in SLN yield. Zhang et al. [38] did not report the yield of SLN, while Piazini et al. [21] reported a yield of approximately 100%; however, the ultrafiltration process was not carried out. In contrast, PN purification involves ultracentrifugation due to the stabilization mechanism caused by steric hindrance [60]. The PVA surfactant acts as a stabilizer and dispersing agent, preventing irreversible aggregation by creating a steric barrier on the particle surface.

**Figure 1:**
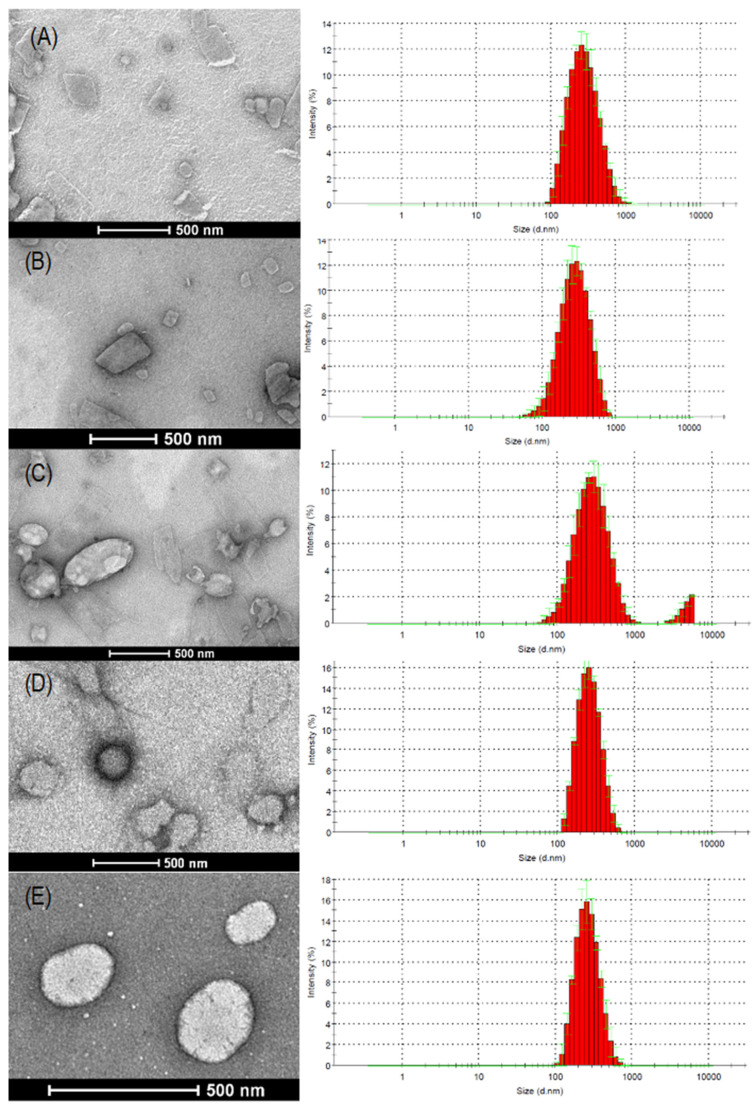
Transmission electron microscopy and particle size histograms obtained by DLS of (**A**) SLN, (**B**) SLN-SIB, (**C**) SLN-SIB-U, (**D**) PN and (**E**) PN-SIB panels.

To observe the morphology of the nanosystems, electron microscopy images were obtained at the nanoscale (Figure 1). For empty SLNs and SLN-SIB, the morphology of the nanoparticles varied from rounded for smaller particles to quadratic or rectangular for larger particles. Another fact to be observed is the presence of only one particle population peak. The addition of URSO to SLN-SIB-U promoted morphological differences in the surface and shape of the nanoparticles; the smaller particles had a rounded shape, the larger ones had an elongated shape, and two population peaks were observed. The more elongated nanoparticles may show increased cellular internalization through enterocytes, which may increase the intestinal absorption of the SLNs prepared with URSO. This fact is due to an increase in the contact surface of the nanoparticles, which increases the cell–nanoparticle interaction through enterocytes [61]. According to Blanco et al. [27], discoidal-shaped nanoparticles are more biodistributed to the hepatic, splenic, and pulmonary compartments. Moreover, SLN-SIB-U showed changes in surface morphology, corroborating the changes in surface charge. These observations indicate that URSO is located on the surface of the particle [62] and that such changes in SLN morphology can improve both intestinal absorption and targeting to liver tissue.

The presence of a bimodal nanoparticle size distribution in SLN-SIB-U could be attributed to a possible excess of URSO not incorporated into the nanosystem. Although the SLN was subjected to an ultrafiltration process with purified water to remove any residual organic solvent or nonencapsulated components, the low aqueous solubility may have prevented the removal of excess URSO, inducing URSO agglomeration at the micromiter scale. The electron microscopy images of PNs and PN-SIB showed spherical particles with a nanoscale diameter consistent with the measurements obtained by DLS. The electron microscopy images of PNs and PN-SIB showed spherical particles with a nanoscale diameter consistent with the measurements obtained by DLS and demonstrated that the presence of SIB did not produce changes in the surface.

#### 3.2.2. SIB Release Studies

**Figure 2:**
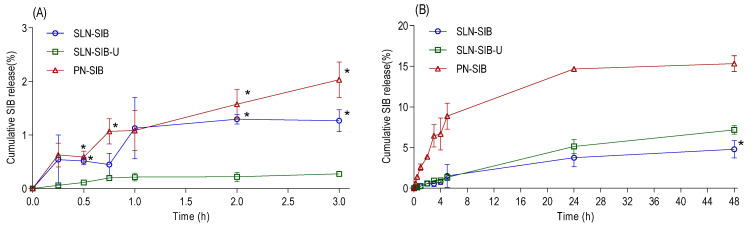
Panel (**A**). In vitro release assay of SLN-SIB. SLN-SIB-U and PN-SIB in SGF at 0.25, 0.5, 0.75, 1.0, 2.0, and 3.0 h. * *p* < 0.05 comparison with SLN-SIB-U. Panel (**B**). In vitro release assay of SLN-SIB. The pH of SLN-SIB-U and PN-SIB in PBS was 7.4 at 0.25, 0.5, 1.0, 2.0, 3.0, 4.0, 5.0, 24, and 48 h. The results are expressed as the mean ± SD, *n* = 3. * *p* < 0.05 in relation to SLN-SIB-U. All SLN-SIB and SLN-SIB-U times showed *p* < 0.05 in relation to PN-SIB.

Release assays were performed in SGF (pH 1.2) and PBS (pH 7.4) for up to 3 h and 48 h, respectively. The SIB release values in SGF (Figure 2A) indicate that SLN-SIB-U provided less release than SLN-SIB and PN-SIB, particularly at 0.5, 2, and 3 h. These release values indicate that the addition of URSO increased the protection of the nanosystem against the gastric environment, corroborating the zeta potential and morphology results. No significant differences were observed in the release profiles of the SLN-SIB and PN-SIB groups (*p* > 0.05).

PN-SIB exhibited greater release in PBS (Figure 2B) than SLN-SIB and SLN-SIB-U. PN-SIB exhibited faster release of SIB under physiological conditions than SLN (*p* < 0.0001). PN-SIB released a total amount of 15.33 ± 0.69, while SLN-SIB and SLN-SIB-U released 4.80 ± 1.07 and 7.17 ± 0.53, respectively. This fact can be unfavorable, as very rapid drug release can occur via rapid elimination. The controlled release of SLN-SIB and SLN-SIB-U may favor a system of sustained release of SIB, maintaining its activity for a longer time at the therapeutic target. Furthermore, SLN-SIB-U showed an initial release profile similar to that of SLN-SIB, except at 24 h and 48 h, when SLN-SIB-U demonstrated greater release, indicating that URSO promotes a slight increase in SLN release [63,64]. According to Palanikumar et al. [64], one of the main reasons for low drug delivery efficiency is the low stability of the encapsulated nanocarrier. This finding indicates that compared with PN-SIB, SLN-SIB-U provides greater stability in terms of SIB encapsulation under both gastric and physiological conditions.

The *J* value and *tlag* value were calculated for SIB encapsulated in nanosystems. Subsequently, mathematical models, including zero-order, first-order, Higuchi, and Korsmeyer–Peppas models, were used to evaluate the kinetic release profile of SIB encapsulated in SLNs or PN based on the highest values of the Pearson correlation coefficient (r^2^) (Table 5). The release of SIB encapsulated in SLN-SIB and PN-SIB was adjusted to the Higuchi model, indicating a controlled release rate of the drug from a matrix system and that drug release is predominantly controlled by the diffusion process. On the other hand, the release of SIB encapsulated in SLN-SIB-U was adjusted to the Korsmeyer–Peppas model, suggesting that the drug is released via Fickian diffusion with distinct release phenomena involving diffusion or swelling [41].

### 3.3. In Vitro Studies in Cell Culture

#### 3.3.1. Cell Proliferation Assay and Viability Assays

**Figure 3:**
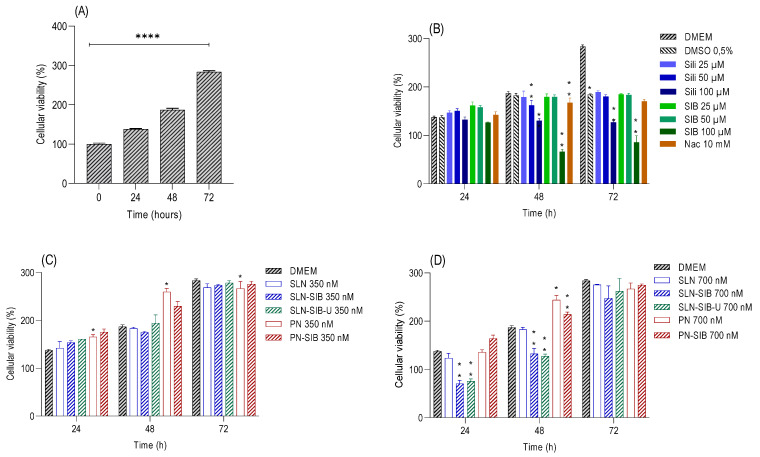
Proliferation assays at 24, 48, and 72 h were performed on the GRX cell line. Panel (**A**). Evaluation of control cell growth at 0, 24, 48, and 72 h. **** *p* < 0.0001. Panel (**B**). Evaluation of pure substances at different concentrations—Sili (25 to 100 µM), SIB (25 to 100 µM), and Nac (10 mM)—in relation to the control DMEM—0.5% DMSO. Panel (**C**). Evaluation of nanosystems at 350 nM in relation to control DMEM and empty nanosystems. Panel (**D**). Evaluation of nanosystems at 700 nM in relation to control DMEM and empty nanosystems. The results are expressed as the mean ± SD, *n* = 4, * *p* < 0.05; ** *p* < 0.05, **** *p* < 0.0001.

**Figure 4:**
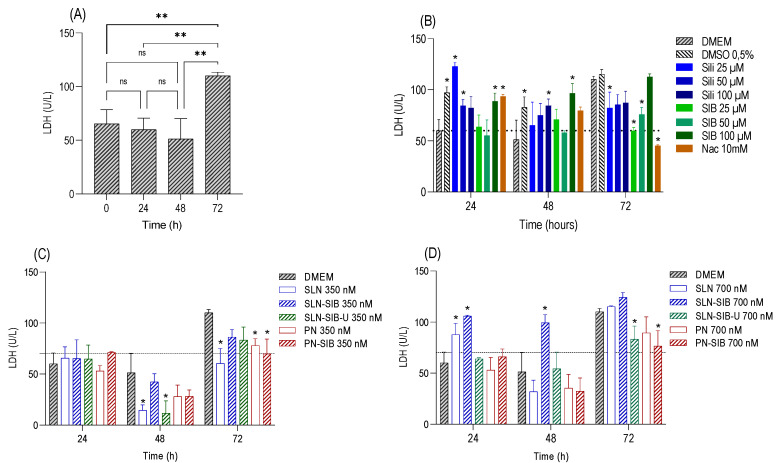
LDH cytotoxicity was measured at 24, 48, and 72 h in the GRX cell line. Panel (**A**). Evaluation of control cell growth at 0, 24, 48, and 72 h. ** *p* < 0.01. Panel (**B**). Evaluation of pure substances at different concentrations—Sili (25 to 100 µM), SIB (25 to 100 µM), and Nac (10 mM)—in relation to the control DMEM (0.5% DMSO). Panel (**C**). Evaluation of nanosystems at 350 nM in relation to control DMEM and empty nanosystems. Panel (**D**). Evaluation of nanosystems at 700 nM in relation to control DMEM and empty nanosystems. The results are expressed as the mean ± SD, *n* = 4, * *p* < 0.05; ns: not significant.

The results of the cell viability assay (Figure S4) revealed that SLNs at a concentration of 1050 nM, Sili at concentrations of 350 µM and 3500 µM, and SIB at a concentration of 3500 µM had cytotoxic effects. Thus, the concentrations selected for the nanosystems were 350 nM and an intermediate concentration of 700 nM. For Sili and SIB, concentrations of 25 µM and 50 µM and an intermediate concentration of 100 µM were selected. The results of the proliferation and LDH assays are shown in Figure 3 and Figure 4, respectively.

The evaluation of the growth of the control DMEM at 0, 24, 48, and 72 h (Figure 3A) demonstrated that there was cell growth between these times through an increase in the cell viability value in relation to time 0. In addition, the LDH levels in DMEM did not significantly differ, except at 72 h, indicating that the cells reached the maximum growth value at that time (Figure 4A).

Thus, the results obtained in the proliferation assay (Figure 3C,D) demonstrated that PN and PN-SIB had greater effects on cell growth than DMEM did after 24 and 48 h at a concentration of 350 nM and after 48 h at a concentration of 700 nM. This finding indicates that the polymer used stimulated the growth of GRX cells. This phenomenon can be attributed to interactions with transmembrane integrins responsible for mechanotransduction [65,66]. According to Christen and Vercesi (2020), PCL can promote the biostimulation of collagen synthesis in the dermis via a mechanotransduction mechanism. As this mechanism promotes the proliferation and biostimulation of collagen synthesis in dermal fibroblasts, the increase in cell proliferation in cells treated with PNs and PN-SIB in relation to that in cells treated with DMEM indicates that mechanotransduction has a biostimulatory effect on GRX myofibroblasts. At 24 and 48 h, 700 nM SLN-SIB and SLN-SIB-U had lower effects on cell growth than SLNs at the same concentration, indicating that SIB had an inhibitory effect on both SLNs. This effect was not observed for Sili or SIB (Figure 3B), which inhibited proliferation after 48 h at a concentration of 100 µM. The effect observed in Sili and SIB is in accordance with the pharmaceutical class of this active compound (class IV), which requires higher concentrations of active compounds to produce the effect [21].

**Figure 5:**
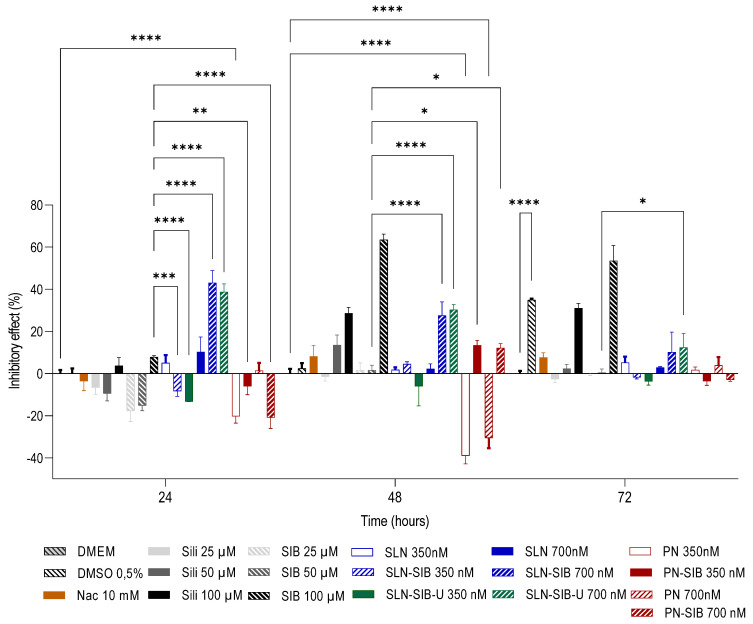
Inhibitory effect of pure substances at different concentrations - Sili (25 to 100 µM), SIB (25 to 100 µM), and Nac (10 mM)- and nanosystems at 350 and 700 nM (SLN-SIB, SLN-SIB-U, and PN-SIB) in relation to their respective controls (0.5% DMSO, SLN, and PN). The results are expressed as the mean ± SD. * *p* < 0.05. ** *p* < 0.005. *** *p* < 0.0005. **** *p* < 0.0001.

Evaluating the effects of the LDH dose on the cytotoxicity of the nanosystems and pure substances (Figure 4) revealed that SLNs and SLN-SIB (700 nM) showed some cytotoxicity at 24 and 48 h, although SLNs did not affect cell growth compared to that in DMEM. However, this phenomenon was not observed for the SLN-SIB-U 700 nM group. This effect may be related to the presence of a higher concentration of stearic acid in SLNs and SLN-SIB than in SLN-SIB-U [67]. Weyenberg et al. [67] verified the cytotoxicity of stearic acid-based SLNs in the J774, 3T3, and HaCaT cell lines. Thus, the incorporation of URSO in SLN-SIB-U 700 nM, in addition to eliminating the cytotoxic effect attributed to stearic acid, maintained the effect of the nanoencapsulated SIB. An inhibitory effect analysis was performed, and the results are shown in Figure 5.

Analysis of the inhibitory effects (Figure 5) produced by all the nanosystems containing SIB and pure substances (Sili, SIB, and Nac) revealed that SLN-SIB and SLN-SIB-U (700 nM) had inhibitory effects similar to those of 100 µM Sili and 100 µM SIB at 48 h. Furthermore, the inhibitory effects of SLN-SIB and SLN-SIB-U (700 nM) at 24 h, in addition to being greater than those of Sili and SIB (100 µM), were similar to those of SLN-SIB and SLN-SIB-U (700 nM) at 24 h, indicating that these effects were maintained at these two time points. In this way, SLN-SIB and SLN-SIB-U have half the effect of SIB at a concentration about 142 times lower. However, despite PN-SIB having some inhibitory effect, the proliferation values of PN and PN-SIB were greater than or similar to, respectively, those of DMEM, indicating that PCL promotes increased proliferation and possibly increased collagen production. Therefore, PN-SIB was excluded from the in vivo assay.

#### 3.3.2. Permeability Assay on Caco-2 Monolayer

**Figure 6:**
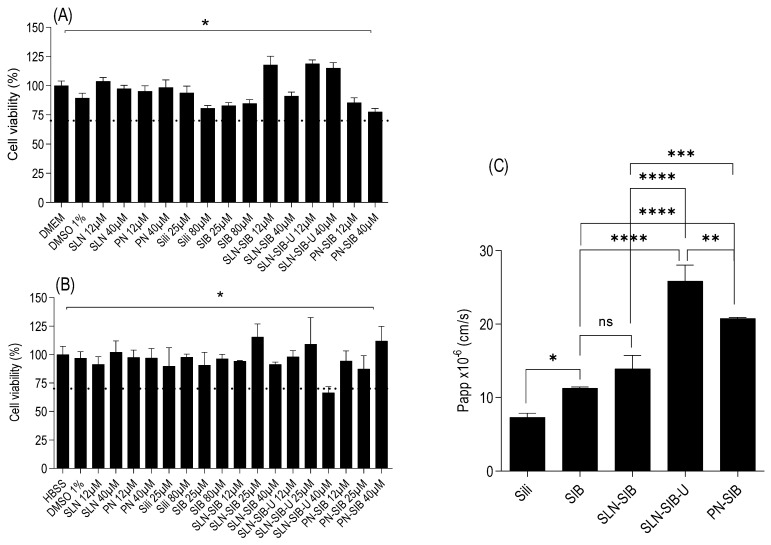
Panel (**A**). Viability of Caco-2 cells cultured in culture medium for 24 h. Panel (**B**). Viability of Caco-2 cells cultured in HBSS for 2 h. * *p*< 0.05 compared to the growth control (DMEM or HBSS). Panel (**C**). Apparent permeability coefficient (*Papp*) of SIB in the Caco-2 monolayer permeability assay. The results are expressed as the mean ± SD. * *p* < 0.05 in relation to Sili. ** *p* < 0.05 in relation to SIB. *** *p* < 0.05 in relation to SLN-SIB. **** *p* < 0.05 compared with SLN-SIB-U.

For the Caco-2 monolayer permeability assay, a cytotoxicity assay was performed in DMEM for 24 h (Figure 6A) and HBSS for 2 h (Figure 6B). This test was carried out with the aim of evaluating the best concentration of drugs and nanoparticles, guaranteeing the integrity of the monolayer during the entire assay. The data obtained from the 24 h cytotoxicity assay showed that only PN-SIB at a concentration of 40 µM had a significant effect on the growth control in DMEM. However, the cell viability was above 70% [68], indicating acceptable cell viability. The HBSS cytotoxicity assay was carried out to simulate the conditions of the Caco-2 monolayer permeability assay. This indicates that a concentration of 40 µM for nanosystems is not recommended for permeability assays. The results of this assay showed that the viability of cells treated with only 40 µM SLN-SIB-U was lower than that of cells treated with DMEM and lower than 70% [68]. Therefore, the use of a concentration of 40 µM is not recommended for permeability assays. At a concentration of 25 µM, no statistically significant differences were observed in relation to DMEM for any of the evaluated nanosystems. With this information, the concentration chosen for the assay was 25 µM.

The results of the Caco-2 monolayer permeability assay (Figure 6C) demonstrated that the *Papp* of SIB was greater than that of Sili, indicating that SIB has greater intestinal permeability than Sili. For the evaluated nanosystems, SLN-SIB presented a *Papp* similar to that of SIB and did not improve permeability. Both SLN-SIB-U and PN-SIB presented *Papp* values greater than those of SIB, particularly SLN-SIB-U, which presented *Papp* values greater than those of PN-SIB and two times greater than those of SIB. This fact can be attributed to the addition of URSO, which acts as a permeation promoter [31].

### 3.4. In Vivo Study

**Figure 7:**
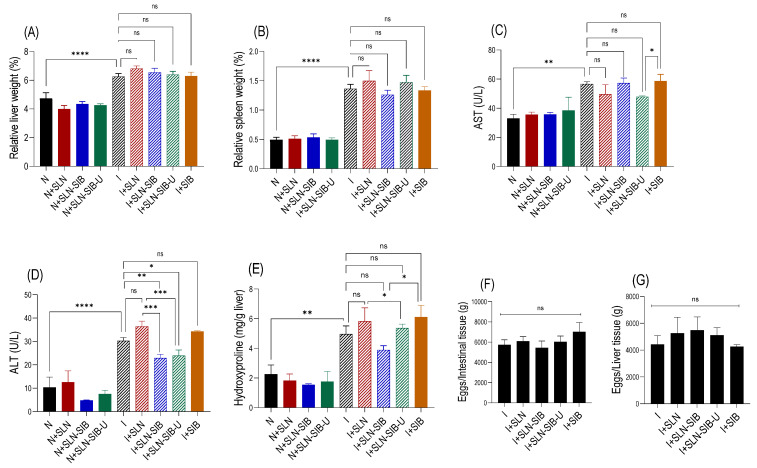
In vivo study results in a BALB/c mice model of *Schistosoma mansoni infection*. Panel (**A**). Relative liver percentage weight in relation to total animal weight. Panel (**B**). Relative spleen percentage weight in relation to total animal weight. Panel (**C**). Aspartate aminotransferase (AST) levels in the serum. Panel (**D**). Alanine aminotransferase (ALT) levels in the serum. Panel (**E**). Biochemical quantification of hydroxyproline. Panel (**F**). Distribution of eggs in intestinal tissue. Panel (**G**). Distribution of eggs in liver tissue. The results are expressed as the mean ± SD; *n* = 3; ns: not significant, * *p* < 0.05, ** *p* < 0.01, *** *p* < 0.001, **** *p* < 0.0001 for the experimental group vs. the I group.

An in vivo study was carried out to investigate the effects of orally administered SIB nanoencapsulated in SLNs (1 mg/kg for 30 consecutive days) on *Schistosoma mansoni* infection in a murine model using BALB/c mice. The in vivo study results are shown in Figure 7.

The parasitological evaluation (Figure 7F,G) demonstrated that the infection was homogeneous among the animals, and there were no significant differences between group I and any of the other treatment groups (I + SIB, I + SLN, I + SLN-SIB, I + SLN-SIB-U). Furthermore, this result demonstrated that the treatment did not interfere with the oviposition of female *Schistosoma mansoni* or with the direction of the eggs to the liver or intestine. This finding corroborates that of Mata-Santos et al. [15], who did not observe differences in the number of eggs in the liver after treatment with Sili. However, the relative liver/spleen weights (Figure 7A,B), liver injury marker AST levels (Figure 7C), and hepatic hydroxyproline levels (Figure 7E) demonstrated that SLN-SIB and SLN-SIB-U did not promote changes in these parameters. This finding differs from that of Mata-Santos et al. [3], who demonstrated a reduction in these parameters by administering Sili for 80 consecutive days at a dose of 10 mg/kg/day intraperitoneally in a murine model of chronic infection caused by *Schistosoma mansoni*. Possible hypotheses for parameters to remain unaltered are differences in the model (acute and chronic phase), therapeutic scheme, or dose used. However, in both the I+SLN-SIB and I+SLN-SIB-U groups, the liver damage marker ALT decreased compared to that in the I and I+SIB groups. This finding differed from that of El-Lakkami et al. [69], who demonstrated that the administration of 750 mg/kg/day Sili orally for 30 days did not change this parameter in relation to that of the infected control group. This information indicates that the hepatoprotective result of SIB nanoencapsulated in SLNs, represented by SLN-SIB and SLN-SIB-U, was greater than that of SIB and Sili at higher doses, which are not able to produce the same effect. This observed effect provides evidence that nanotechnology has improved the oral bioavailability of SIB.

### 3.5. Stability Study

**Figure 8:**
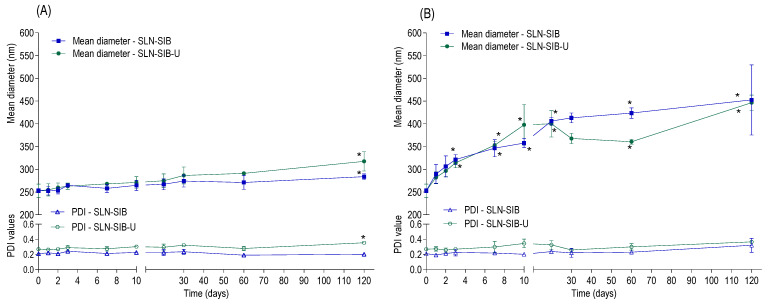
Stability of SLN-SIB and SLN-SIB-U in terms of mean diameter and polydispersity index (PDI) evaluated at 0, 1, 2, 3, 7, 10, 20, 30, 60, and 120 days. Panel (**A**). Stability under refrigeration (4 ± 0.2 °C). Panel (**B**). Stability at room temperature (25 ± 0.5 °C). The results are expressed as the mean ± SD, *n* = 3, * *p* < 0.05 in relation to time 0.

The stability study was carried out in a refrigerator (4 ± 0.2 °C) and at room temperature (25 ± 0.5 °C) for 120 days for SLN-SIB and SLN-SIN-U (Figure 8).

For the stability evaluations for SLN-SIB and SLN-SIB-U in the refrigerator, there was a small change in the mean diameter and PDI at the end of 120 days; however, these values remained at approximately 300 nm and 0.3, respectively, which is consistent with the findings of other authors [21,27,28,48,52]. This information indicates that both formulations were stable under refrigeration (4 ± 0.2 °C) for a period of 120 days. According to the stability evaluations of SLN-SIB and SLN-SIB-U at room temperature, large differences in the mean diameter were observed from day 3, with a significant increase in size (>300 nm), indicating the possible beginning of particle aggregation and loss of formulation stability. These results highlight the relevance of identifying critical parameters impacting the manufacturing and use of nanoparticle-based formulations. An evaluation of the optimal storage conditions for SLNs to assess the long-term activity of the formulations must be performed so a comprehensive characterization of the nanosystems is achieved [70].

## 4. Conclusions

The use of nanotechnology to improve the oral bioavailability of SIB is a promising alternative due to the in vitro and in vivo results presented in this study. SLN-SIB, SLN-SIB-U, and PN-SIB nanosystems with sizes less than 260 nm and an encapsulation efficiency higher than 75% were successfully prepared, and characterized electron microscopy images revealed spherically shaped particles that may favor their pharmacokinetic properties. A proliferation assay with the GRX cell line revealed that inhibitory effects of approximately 43 and 39 were obtained for SLN-SIB and SLN-SIB-U, respectively, whereas PN-SIB did not show an inhibitory effect. Furthermore, SLN-SIB-U showed a greater apparent permeability coefficient than PN-SIB, SLN-SIB, and pure SIB. These data suggest that lipid-based nanosystems are promising candidates for in vivo studies. In vivo studies performed with a murine model of acute-phase *Schistosomiasis mansoni* infection revealed that SLN-SIB and SLN-SIB-U can have hepatoprotective effects, as indicated by lower ALT values than those in controls. Although SLNs were kept refrigerated before characterization, this study, like many others, has limitations associated with a lack of data demonstrating the size stability of SLNs during cell culture and in vivo assessment.

In conclusion, SLN-SIB-U represents a promising nanosystem for enhancing the pharmacokinetic properties of SIB considering the 3Rs approach. However, more studies are needed to advance to the next stages of development of this nanosystem as a new pharmaceutical formulation to enable its use as a complementary treatment or for the treatment of sequelae of *Schistosomiasis mansoni* infection. In addition, other pharmacokinetic and pharmacodynamic studies can be explored to investigate the observed effects of silybin in this nanosystem on other liver diseases.

## Figures and Tables

**Table 1:** Preparation conditions and composition of lipid nanoparticles.

Nanoparticle	Method	Preparation Conditions		Composition			
		UF	US (min)	P20 (% *w*/*v*) ^1^	Stearic Acid (mg) ^2^	SIB (mg) ^2^	URSO (mg) ^2^
NP-1	A	No	30	1.30	210	10	-
NP-2	B	No	30	1.67	210	10	-
NP-3	C	No	40	1.67	210	10	-
NP-4	D	No	50	1.67	210	10	-
NP-5	D	No	50	1.67	200	10	10
NP-6	D	No	50	1.67	190	10	20
NP-7	D	No	50	1.67	180	10	30
NP-8	D	No	50	1.67	160	10	50
SLN	D	Yes	50	1.67	210	-	-
SLN-SIB	D	Yes	50	1.67	210	10	-
SLN-SIB-U	D	Yes	50	1.67	160	10	50

Legend: UF: ultrafiltration; US: ultrasonication; ^1^ aqueous phase; ^2^ organic phase; P20: polysorbate 20; SIB: silybin; URSO: ursodeoxycholic acid.

**Table 2:** The experimental animal groups were treated orally for 30 days.

Animal Groups	Infection	Dose (mg·kg^−1^)
N *	No	-
N + SLN	No	1
N + SLN-SIB	No	1
N + SLN-SIB-U	No	1
I *	Yes	-
I + SIB	Yes	10
I + SLN	Yes	1
I + SLN-SIB	Yes	1
I + SLN-SIB-U	Yes	1

* Control; N: noninfected; I: infected.

**Table 3:** Physicochemical characterization of SLNs obtained using different preparation methods.

Nanoparticle	Method	Mean Diameter (nm)	PDI	Zeta Potential (mV)
NP-1	A	426.7 ± 48.3	0.428 ± 0.01	−12.1 ± 1.8
NP-2	B	386.8 ± 19.0	0.258 ± 0.02	−11.7 ± 1.7
NP-3	C	291.8 ± 16.1	0.206 ± 0.03	−12.6 ± 2.2
NP-4	D	260.9 ± 4.5	0.197 ± 0.02	−11.6 ± 0.9
NP-5	D	242.1 ± 1.4	0.178 ± 0.01	−11.1 ± 0.2
NP-6	D	251.4 ± 14.5	0.209 ± 0.03	−12.0 ± 0.8
NP-7	D	288.8 ± 10.8	0.250 ± 0.03	−11.0 ± 1.9
NP-8	D	279.2 ± 14.0	0.252 ± 0.03	−12.1 ± 2.1
SLN	D	221.2 ± 9.7	0.154 ± 0.01	−30.9 ± 1.8
SLN-SIB	D	252.8 ± 4.4	0.209 ± 0.01	−34.5 ± 2.3
SLN-SIB-U	D	252.9 ± 14.4	0.269 ± 0.01	−27.3 ± 1.3

Results are expressed as the mean ± standard deviation.

**Table 4:** Physicochemical characterization of the selected SLNs and PNs.

Nanoparticle	Mean Diameter (nm)	PDI	Zeta Potential (mV)	EE (%)		Yield (%)
				SIB	URSO	
SLN	221.2 ± 9.7	0.154 ± 0.005	−30.9 ± 1.8	-	-	-
SLN-SIB	252.8 ± 4.4 *	0.209 ± 0.007 *	−34.5 ± 2.3 *	90.3 ± 2.2	-	38.8 ± 6.0
SLN-SIB-U	252.9 ± 14.4 *	0.269 ± 0.005 *	−27.3 ± 1.3	77.1 ± 2.8	92.55 ± 4.3	32.1 ± 4.8
PN	267.3 ± 4.6	0.178 ± 0.045	−2.1 ± 0.4	-	-	-
PN-SIB	241.8 ± 4.1 **	0.139 ± 0.017	−2.2 ± 0.2	98.0 ± 0.2	-	86.8 ± 5.5

The results are expressed as mean ± standard deviation; PDI: polydispersity index; EE: encapsulation efficiency; SIB: silybin; URSO: ursodeoxycholic acid. * (*p* > 0.05) in relation to the control/empty nanoparticle; ** (*p* > 0.01) in relation to the control/empty nanoparticle.

**Table 5:** Kinetic release analysis using mathematical models, flow through membrane (*J*), and lag time (*tlag*) values of the SIB release profile from nanosystems.

Nanosystems	SLN-SIB		SLN-SIB-U		PN-SIB	
Medium	SGF	PBS	SGF	PBS	SGF	PBS
*Kinetic model* *						
Zero-order	0.686	0.925	0.708	0.914	0.958	0.885
First-order	0.640	0.563	0.562	0.687	0.867	0.532
Korsmeyer–Peppas	0.663	0.966	0.835	0.919	0.908	0.926
Higuchi	0.730	0.981	0.813	0.839	0.962	0.939
*J* (µg/h^−1^)	16.58 ± 3.79	4.65 ± 0.99	2.96 ± 0.74	5.88 ± 0.36	28.94 ± 4.90	11.49 ± 2.52
*tlag* (h)	0.95 ± 0.72	2.91 ± 1.65	0.93 ± 0.43	2.03 ± 1.32	0.58 ± 0.05	7.65 ± 2.48

SGF: simulated gastric fluid, pH 1.2; PBS: phosphate-buffered saline, pH 7.4; * Pearson correlation coefficient (r^2^).

## Data Availability

The data are contained within the article and supplementary materials.

## Supplementary Materials

The following supporting information can be downloaded at: https://www.mdpi.com/article/10.3390/pharmaceutics16050618/s1, Figure S1: Chemical structures of (A) silybin and (B) ursodeoxycholic acid; Figure S2: Pareto chart (A), superficial response of ultrasonication x surfactant graph (B), superficial response of ursodeoxycholic acid addition x surfactant graph (C), and superficial response of ursodeoxycholic acid addition x ultrasonication graph (D) in relation to the particle size parameter; Figure S3: Pareto chart (A), superficial response of ultrasonication x surfactant graph (B), superficial response of ursodeoxycholic acid addition x surfactant graph (C), and superficial response of ursodeoxycholic acid addition x ultrasonication graph (D) in relation to the PDI parameter; Figure S4: Analysis of cell viability in the GRX cell line within 24 h using the SLN (A), PN (B), Sili and SIB (C) MTT assays.
