# Supplementary material for: Evaluation of Silybin Nanoparticles against Liver Damage in Murine Schistosomiasis mansoni Infection
Source: Pharmaceutics. 2024 May 4;16(5):618. doi: 10.3390/pharmaceutics16050618 (PMC11125168; doi:10.3390/pharmaceutics16050618)
Supplement: Supplementary file 1 [file pharmaceutics-16-00618-s001.zip › pharmaceutics-2963560-supplementary.pdf]

SUPPLEMENTARY MATERIALS

Supplementary information (section 3.)

Chemical structure of silybin and ursodeoxycholic acid

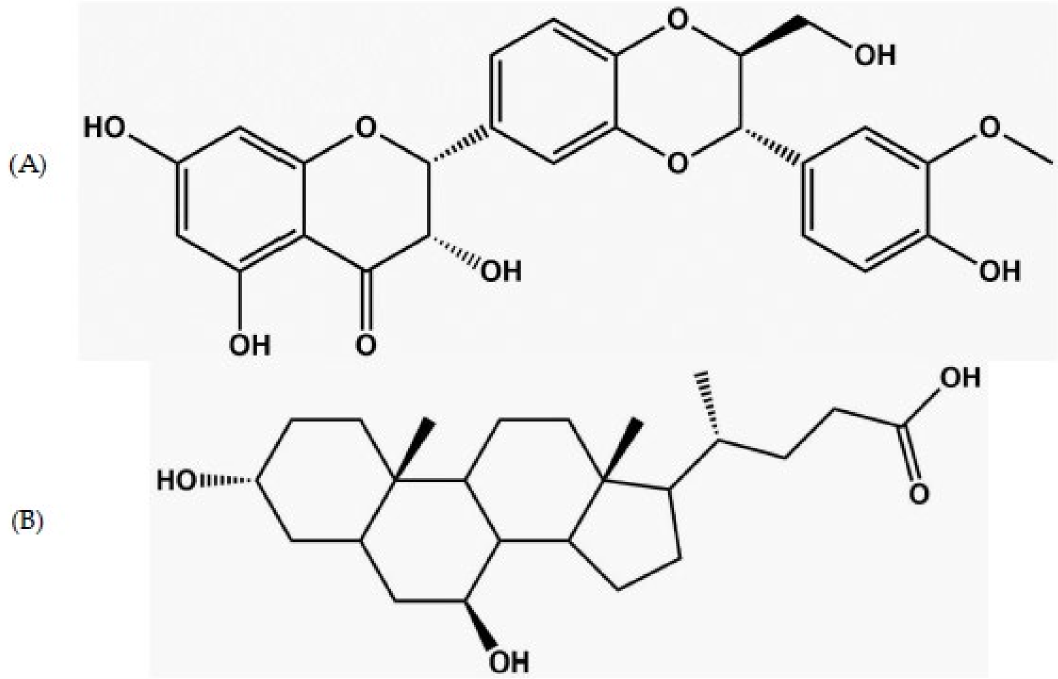

Figure S1. Chemical structures of silybin (A) and ursodeoxycholic acid (B).

Impact of modifications in nanoparticle preparation using Pareto analysis

The impact of modifications to the method and/or composition of solid lipid nanoparticles (SLN) containing silybin on the particle size parameters was evaluated, as illustrated in Figure S2. The Pareto chart (Figure S2A) revealed a significant impact resulting from an increase in sonication time, which was inversely proportional to the particle size. The response surface plots depict, within the green regions, the formulations that achieved the smallest particle sizes. These formulations are associated with changes in specific variable sets of sonication time vs. surfactant addition (Figure S2B), ursodeoxycholic acid addition vs. surfactant (Figure S2C), and ursodeoxycholic acid addition vs. sonication time (Figure S2D).

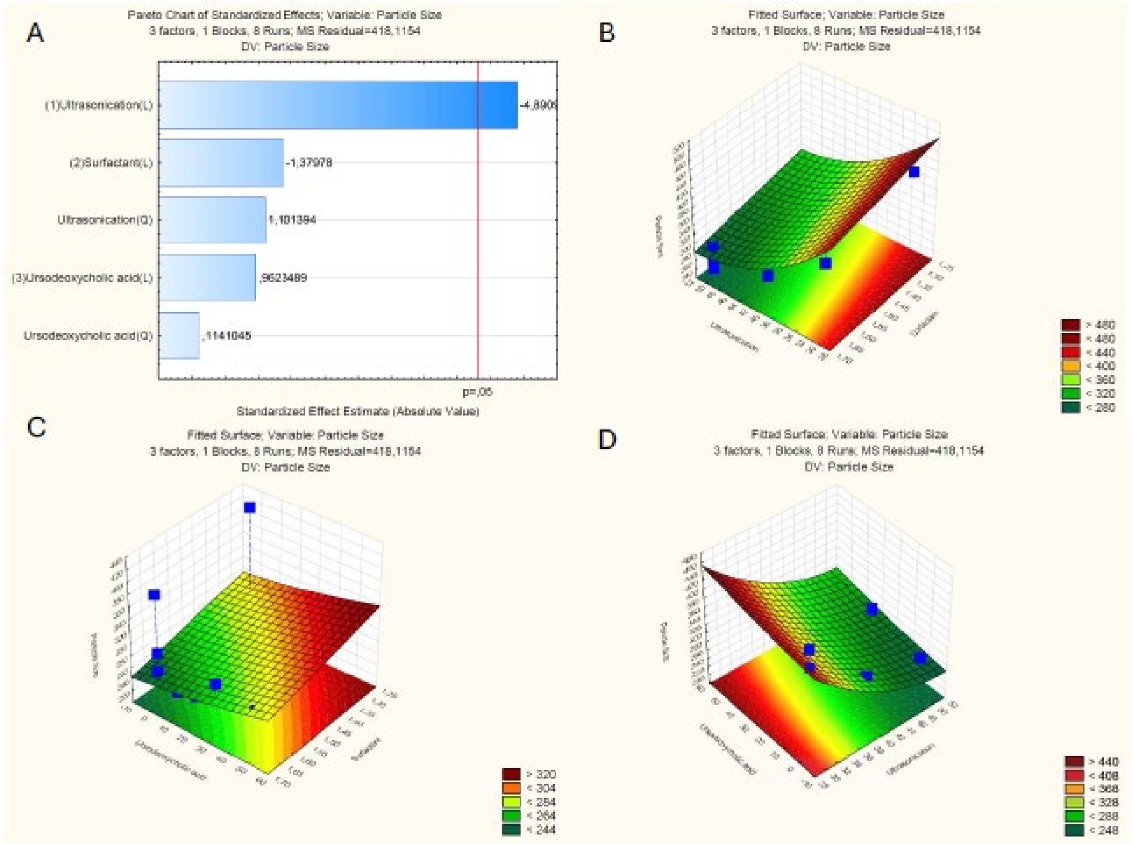

Figure S2. Pareto chart (A), superficial response of ultrasonication x surfactant graph (B), superficial response of ursodeoxycholic acid addition x surfactant graph (C), and superficial response of ursodeoxycholic acid addition x ultrasonication graph (D) in relation to the particle size parameter.

Furthermore, the influence of modifications in the method and/or composition of SLN containing silybin was investigated with respect to the polydispersity index (PDI) parameter, as demonstrated in Figure S3. The Pareto diagram (Figure S3A) revealed that an increase in the amount of surfactant had an inversely proportional relationship with the polydispersity index. The response surface plots within the green regions depict the formulations that present the smallest PDI values. These formulations are related to changes in specific variable combinations of sonication time vs. surfactant addition (Figure S3B), ursodeoxycholic acid addition vs. surfactant (Figure S3C), and ursodeoxycholic acid addition vs. sonication time (Figure S3D).

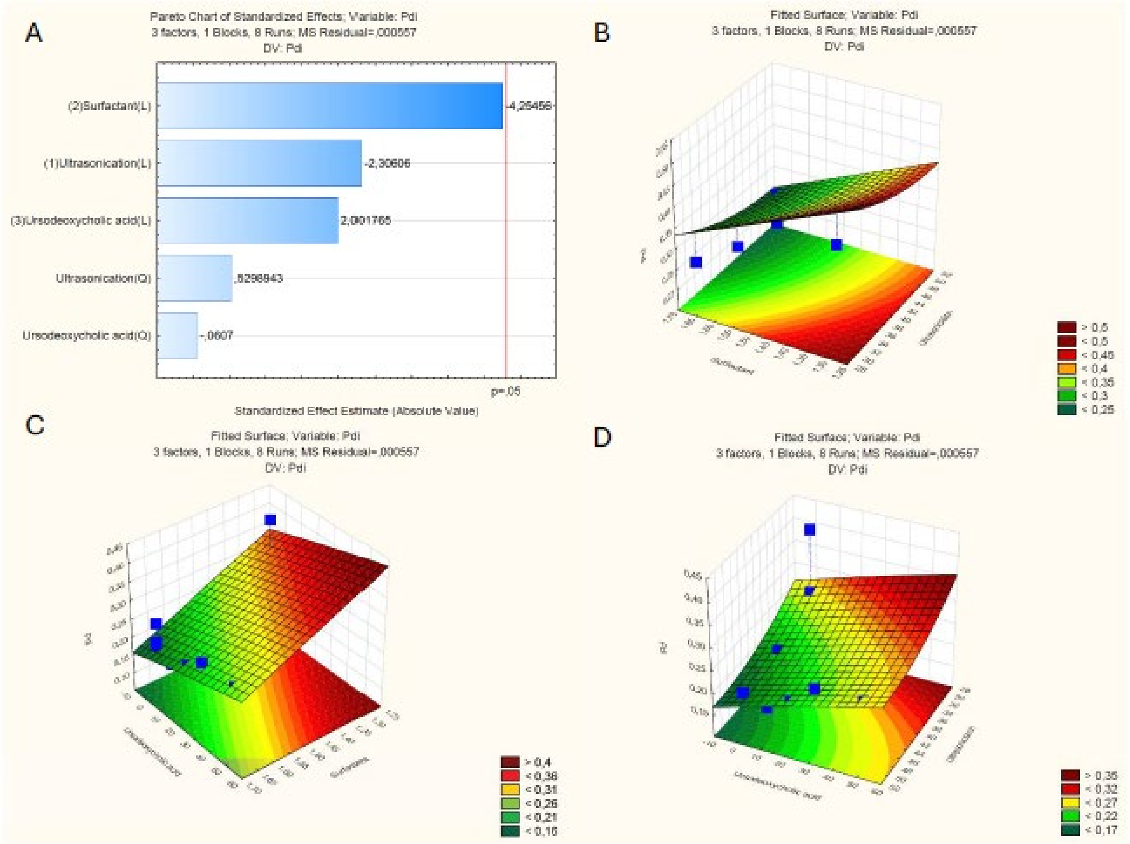

D
C
B
A

Figure S3. Pareto chart (A), superficial response of ultrasonication x surfactant graph (B), superficial response of ursodeoxycholic acid addition x surfactant graph (C), and superficial response of ursodeoxycholic acid addition x ultrasonication graph (D) in relation to the PDI parameter.

Cell viability assay

Before the proliferative assay, the cell viability assay was performed using 3-(4,5-dimethyl-2-thiazolyl)-2,5-diphenyl-2H-tetrazolium bromide (MTT) to investigate the concentrations to be used in the cell proliferation assay. For this purpose, $3x10_4$ cells per GRX well were seeded in 96-well plates and incubated for 24 hours for cellular adhesion. After this period, the cells were incubated with nanoparticles or controls for 24 hours. For this assay, concentrations of 0.35, 3.5, 35, 350, and 3500 µM Sili and SIB were used. In the preparation of Sili and SIB, at all concentrations, a stock solution in dimethyl sulfoxide (DMSO) was prepared, and aliquots were diluted in complete medium to obtain the desired concentrations of substances and 0.5% DMSO. To prepare the nanosystems, SLN-SIB, SLN-SIB-U, and PN-SIB were diluted to obtain concentrations of 3.5, 35, 350, 1050, 3500, and 35000 nM, respectively, as well as their controls (SLN and PN) at the same concentrations. All concentrations of nanosystems were diluted in complete medium. The cell viability

assay consisted of mitochondrial cleavage of MTT tetrazolium salt (0.5 mg/mL) incubated for 3 h, which culminates in the
formation of formazan crystals, quantifying the cell viability from mitochondrial activity. DMSO was used to dissolve these crystals, and quantification was performed with a spectrophotometer (SpectraMax® I3, Molecular Devices, San Jose, California) at an absorbance of 570 nm. The analysis of cell viability in the GRX cell line of the nanosystem and pure substance using MTT assays was shown in Figure S4.

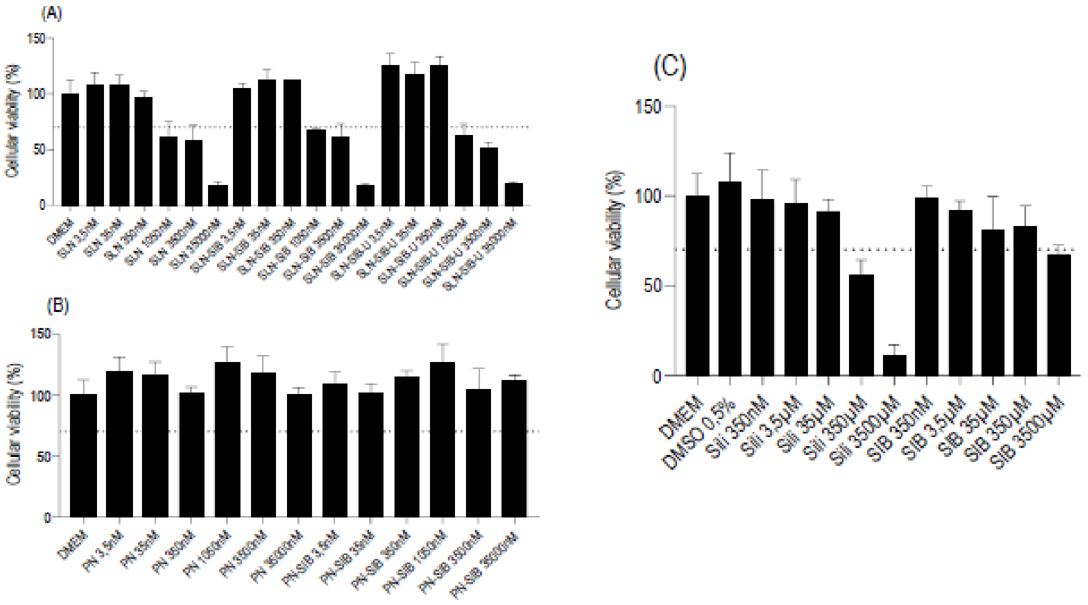

Figure S4. Analysis of cell viability in the GRX cell line within 24 hours of the SLN (A), PN (B), Sili and SIB (C) using MTT assays.
